# Feasibility and efficacy of peak-velocity interval training vs. moderate-intensity walking training in people with multiple sclerosis with severe fatigue and walking impairment: A pilot randomized controlled trial

**DOI:** 10.1016/j.msard.2025.106930

**Published:** 2025-12-12

**Authors:** Brice Thomas Cleland, Brenda Jeng, Natalya Brown, Robert W. Motl, Sangeetha Madhavan

**Affiliations:** a Brain Plasticity Laboratory, Department of Physical Therapy, College of Applied Health Sciences, University of Illinois Chicago, Chicago, IL, USA; b Exercise Neuroscience Research Laboratory, Department of Kinesiology and Nutrition, College of Applied Health Sciences, University of Illinois Chicago, Chicago, IL, USA

**Keywords:** Multiple sclerosis, Rehabilitation, Gait, Treadmill training, High intensity

## Abstract

**Introduction:**

High-intensity interval training (HIIT) may yield greater improvements in walking and fatigue for people with multiple sclerosis (MS) than moderate-intensity, continuous training (MICT). This pilot project established the feasibility of peak velocity interval walking training (PVIT, a novel form of HIIT) in people with MS who had elevated fatigue and walking dysfunction and determined initial efficacy on fitness, walking, fatigue, and cognition.

**Methods:**

Twelve people with MS [49.3 (7.4) years of age; 9 female; 1–34 years post diagnosis; Fatigue Severity Scale Score >4; Patient-Determined Disease Steps score 3–6] were randomly assigned into PVIT (*n* = 7) or MICT (*n* = 5) and completed 12 sessions of training up to 40 min. Feasibility was measured throughout as rates of recruitment, randomization, retention, adherence, and compliance. Pre- and post-assessments included peak oxygen consumption, walking performance, fatigue severity, and cognition.

**Results:**

All participants who enrolled were successfully randomized and completed all sessions (100 % retention) with no adverse events. Adherence was high (86 %) as was intensity compliance (100 %) and did not differ between groups. PVIT resulted in greater walking velocity (relative to maximal overground velocity, 95 % vs. 70 %) and heart rate reserve (93 % vs. 55 %) than MICT. Peak oxygen consumption improved more in the PVIT than MICT condition (14 % vs. 1 % improvement, *p* < 0.05).

**Conclusion:**

Initial results suggest that PVIT as a form of HIIT is safe, feasible, and may improve aerobic fitness more than MICT in people with MS who have elevated fatigue and impaired walking function.

**Clinical trial registration:**

ClinicalTrials.gov: NCT06264336

## 1. Introduction

Multiple sclerosis (MS) is an immune-mediated, neurodegenerative disease of the central nervous system that affects ~1 million adults in the US (Compston and Coles, 2008; Wallin et al., 2019). Hallmark features of MS (>50 % prevalence) include walking impairment, symptomatic fatigue, and cognitive deficits (Chiaravalloti and DeLuca, 2008; Green et al., 2017; Krupp, 2003; Motl et al., 2010; Weiland et al., 2015). These features of MS are inter-related; symptomatic fatigue often drives declines in walking, cognitive impairment, and worsening disability (Huisinga et al., 2011; Motl et al., 2011; Young et al., 2021), which compromise quality of life and independence (Green et al., 2017; Janardhan and Bakshi, 2002; Krupp, 2003; Krupp and Elkins, 2000). Walking dysfunction increases the metabolic cost of walking, resulting in early onset of fatigue or elevated fatigue (Motl et al., 2011). These three symptoms may cause secondary reductions in physical activity, aerobic capacity, quality of life, and independence (Janardhan and Bakshi, 2002; Langeskov-Christensen et al., 2015; Ritvo et al., 1996; Schwartz et al., 1996). However, these symptoms are poorly managed with conventional disease-modifying medications or rehabilitation therapy (Brichetto et al., 2003; Heine et al., 2015; Jones et al., 2025; Miller and Soundy, 2017; Morrow, 2024; Panitch and Applebee, 2011; Pearson et al., 2015; Snook and Motl, 2009; Zielińska-Nowak et al., 2020).

Exercise training is one potential approach for improving walking, fatigue, and cognition in MS (Halabchi et al., 2017). People with MS are recommended to engage in regular low-to-moderate-intensity exercise (30 min/day on 2 days/week) (Myers et al., 2010), which is often achieved through moderate-intensity, continuous training (MICT). Although MICT enhances aerobic capacity, mobility, strength, cognition, and quality of life (Halabchi et al., 2017; Heine et al., 2015; Langeskov-Christensen et al., 2015; Latimer-Cheung et al., 2013; Pilutti et al., 2013), improvements in walking function, symptomatic fatigue, and cognition have been small, bringing the efficacy of this approach into question (Gharakhanlou et al., 2021; Pearson et al., 2015; Snook and Motl, 2009).

In people without neurological impairment, high-intensity, interval-based training (HIIT) leads to greater improvements in walking endurance, mobility, and brain plasticity than MICT (Andrews et al., 2020; Coetsee and Terblanche, 2017). In people with MS, a small number of studies reported that high-intensity aerobic training improves walking speed, endurance, aerobic capacity, and muscle strength and power (Bae and Kasser, 2023; Campbell et al., 2018). When compared to MICT, a systematic review reported that HIIT led to greater improvements in aerobic capacity and cognition (memory) than MICT in MS (Youssef et al., 2024).

Despite the potential benefits of HIIT, there are several prominent field-wide limitations of research on exercise training, walking outcomes, and fatigue in MS. First, researchers have primarily enrolled people with MS with no or mild walking disability, and low or unreported levels of symptomatic fatigue (Latimer-Cheung et al., 2013; Martin-Nunez et al., 2025). Both of those enrollment choices yield floor effects in outcomes and prevent conclusions on exercise as a treatment approach for walking and fatigue (Andreasen et al., 2011). Second, nearly all previous studies of HIIT have used resistance training, cycle ergometers, or recumbent steppers, not walking training, as the exercise modality (Bae and Kasser, 2023; Campbell et al., 2018; Martin-Nunez et al., 2025; Youssef et al., 2024). Although these modalities yield benefits, the principle of specificity suggests that exercise interventions that involve walking are critical for improving walking, independence, and community involvement in people with MS. HIIT with a walking modality provides a greater stimulus than MICT for improving outcomes in other neurological populations (e.g., stroke), but this approach has not been researched in MS (Boyne et al., 2016; Dalgas et al., 2019).

This project aimed to overcome those field-wide limitations by enrolling people with MS with walking dysfunction and symptomatic fatigue and providing high-intensity exercise training with a walking modality, specifically peak velocity interval training (PVIT) (Madhavan et al., 2025). PVIT is a novel stimulus that focuses on achieving peak walking velocities in populations with walking dysfunction and allows for walking velocities that elicit heart rates satisfying criteria for “high intensity.” The aims of this project were to evaluate the feasibility of PVIT (randomization, retention, adherence, and compliance) and assess the effects on fitness, walking, symptomatic fatigue, and cognition compared to a traditional MICT approach in an underserved MS population.

## 2. Methods

### 2.1. Participants

This study was approved by the University of Illinois Chicago institutional review board (STUDY2023-1555, 01/2024) and conformed to the ethical standards of the Declaration of Helsinki. All participants provided written, informed consent. This clinical trial was prospectively registered (ClinicalTrials.gov: NCT06264336). Data were collected from March 2024 through February 2025 in the Brain Plasticity Laboratory at the University of Illinois Chicago . Individuals >21 years of age were included if they had a diagnosis of MS, stable disease-modifying therapy over the preceding 6 months, walking dysfunction, symptomatic fatigue, and ability to walk for 6 min at self-paced speed (assistive device accepted). To define walking dysfunction, we only included individuals with a Patient-Determined Disease Steps (PDDS) (Hohol et al., 1995; 1999) score of 3–6, which includes people with some gait disability to those needing bilateral support. This range excludes people with no impact of MS on walking and those unable to walk 25 ft. even with support. To define symptomatic fatigue, we only included individuals with a Fatigue Severity Scale (FSS) (Krupp et al., 1989) score ≥ 4, as outlined in the initial publication. Finally, we only included people who could walk for 6 min at self-paced speed (assistive device accepted) to ensure that we could provide a minimal exercise stimulus (warmup and start of an interval). Individuals were excluded if they had a relapse in the preceding 30 days, other neurological diseases besides MS, uncontrolled cardiorespiratory or metabolic diseases, cognitive impairment (Mini Mental State Examination [MMSE] score <23), severe osteoporosis, or unable to pass a graded exercise stress test. Data for this study are openly available at: doi.org/10.25417/uic.28661129.

This was a single-center, parallel-groups randomized controlled trial. Group assignment was randomly determined (no stratification) with a random number generator and concealed from investigators performing outcomes assessment. Blinding was not possible for trainers. Although blinding was not technically possible for participants, they were not explicitly informed about which group they were assigned. Regardless of training group, all participants performed 12 training sessions (2–3 sessions/week). At the start of each week, maximal walking velocity was assessed with the 10-meter walk test (10MWT). Treadmill sessions started (warmup) and ended with 5 min of walking at 50 % of the weekly maximal walking velocity. Prescribed walking time per session was 40 min (inclusive of any pauses with the treadmill stopped). Peak heart rate (HR), velocity, and distance walked were recorded. For safety, participants wore a harness without body-weight support and were allowed to hold onto handrails as necessary.

The planned sample size was 20 participants (10 per group). Because this was a pilot project, this sample size was selected to obtain important information about the safety and feasibility of high-intensity exercise training with a walking modality while remaining within the confines of a limited budget. Additionally, this sample size was selected based on data from Wens et al. 2015, which showed an increase in VO_2_ peak of ~4.1 mL/kg/min across a training period in people undergoing high intensity interval aerobic training (Wens et al., 2015). A sample size of at least 5 would be needed to achieve a similar effect size (*d* = 1.9). We performed an interim analysis after enrolling the 12 participants presented here and found that we had achieved a medium between group effect size for change in VO_2_ peak, and the pilot study was stopped to conserve resources.

### 2.2. Peak velocity interval training (PVIT)

**Fig. 1.**
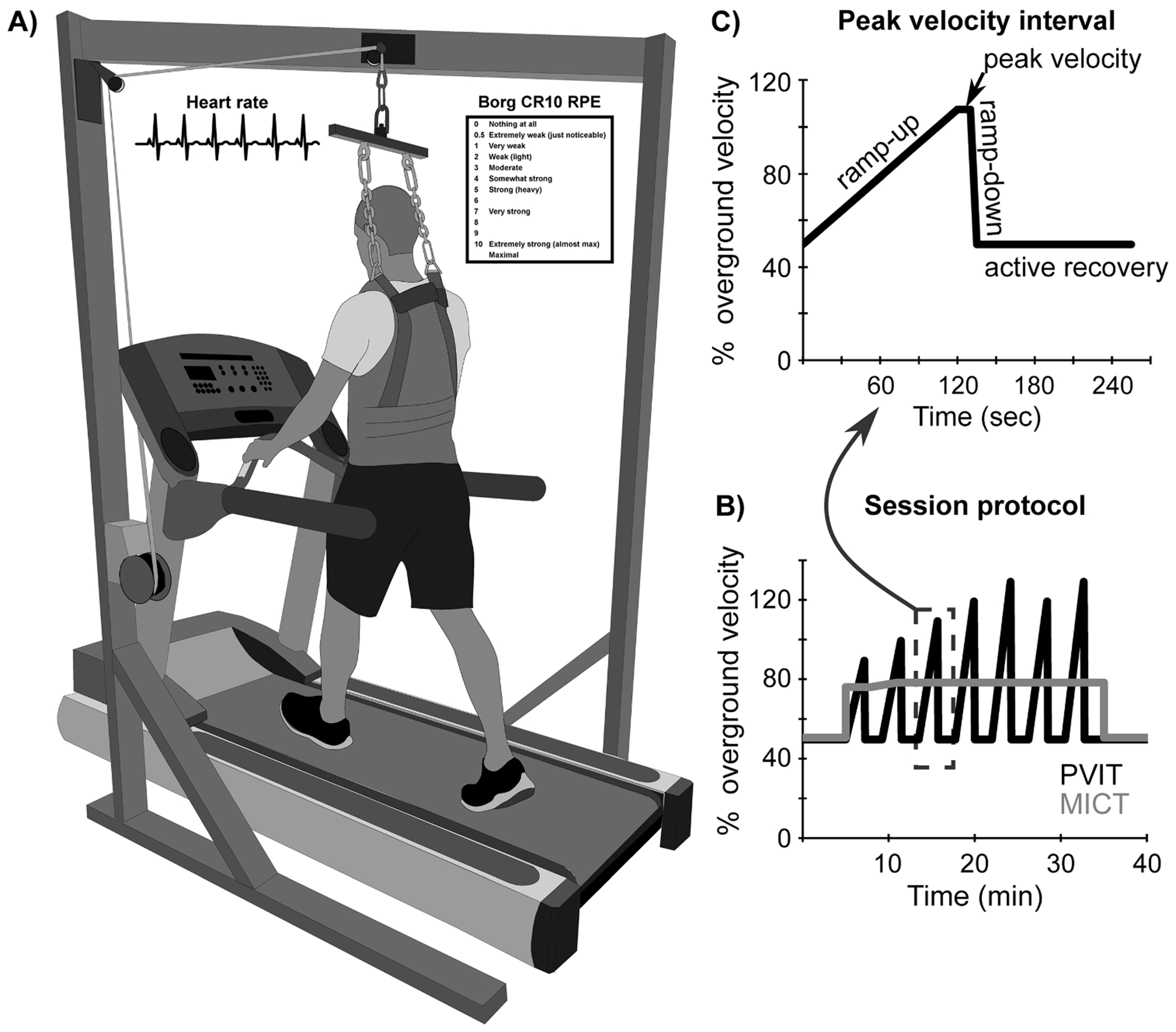
Treadmill setup and PVIT protocol. A) Participants walked on a treadmill with a safety harness (no bodyweight support). Heart rate and Rating of Perceived Exertion (RPE, Borg 0–10 scale) were measured. B) The peak velocity interval training (PVIT) group performed alternating intervals of walking at peak velocity and active rest at 50 % of maximal overground velocity. The moderate-intensity continuous training (MICT) group performed walking at a moderate intensity (based on heart rate reserve). C) In the PVIT group, peak velocity intervals involved a ramp up period over the course of two minutes up to the maximal velocity that could be safely maintained for 10 s, followed by a ramp down at least 2 min of active recovery.

After warmup, the PVIT group performed peak velocity intervals as described previously (Fig. 1) (Madhavan et al., 2025, 2019). For each interval, treadmill velocity was increased over a 2-minute period up to the peak velocity that was safe and tolerable. Peak velocity was held for 10 s. After each interval, participants walked at their warm-up, recovery velocity until HR was within 5 bpm of warmup. If HR did not decrease within 4 min, the treadmill was stopped, and participants stood until HR reached the required level. At the end of intervals and recovery periods, Rating of Perceived Exertion (RPE; Borg 10-point scale) was assessed. Across training sessions, peak treadmill velocity was continuously increased. If the participant could safely maintain the peak velocity achieved during an interval, treadmill velocity was increased for the subsequent interval. At velocities below 3.3 mph (1.48 m/s), each increase in peak speed was 5–10 % of the previous peak velocity. To avoid transition to running, increases in peak velocity were relatively smaller when increasing above 3.3 mph. If participants displayed foot dragging, needed excessive manual assistance, or displayed other signs of instability, peak velocity was decreased by 5–10 % for the subsequent interval.

### 2.3. Moderate-intensity continuous training (MICT)

After warmup, the MICT group walked at a speed eliciting a HR of 40–60 % of HR reserve (peak HR – resting HR) (Halabchi et al., 2017). HR was continuously monitored and RPE was assessed every 3 min. Within and across the 12 training sessions, peak treadmill velocity was increased or decreased to maintain HR within the prescribed HR range.

### 2.4. Outcomes

Outcome measurements were performed before and after completion of the 12 training sessions across 2 visits within 7 days of the start/end of training.

#### 2.4.1. Feasibility

- **Process feasibility:** Recruitment rate was measured as the proportion of people approached for screening who were enrolled. Randomization rate was measured as the proportion of enrolled people who were randomized to a group.
- **Resource feasibility:** Retention rate was measured as the proportion of enrolled and randomized participants who completed the study. Adherence was measured as the percentage completion of originally scheduled sessions (sessions not cancelled or rescheduled). Compliance was measured as the percentage of sessions complying with the prescribed exercise [no modifications to session duration (duration compliance) or intensity (intensity compliance)].
- **Intervention fidelity:** The percentage of datapoints that met HR criteria for high-intensity exercise for the PVIT group [HR: 60+ % heart rate reserve (HRR)] and moderate-intensity exercise for the MICT group (HR: 40–60 % HRR) was calculated.

#### 2.4.2. Aerobic power

- **Peak rate of oxygen consumption (VO_2_ peak).** A graded cardiopulmonary exercise stress test was performed on a treadmill. The fastest comfortable walking speed was determined during a 3–5 min warmup at 0 % grade. During the test, this speed was held constant, and grade was progressively increased by 2 % every 2 min. An electrocardiogram was continuously monitored, and blood pressure (BP), RPE, and oxygen saturation were assessed at the end of each 2-minute stage. Resting and peak HR were measured. Expired gases were collected and analyzed using an open-circuit spirometry system (Quark, COSMED USA Inc., IL, USA) to quantify VO_2_ peak. The test was terminated when the participant reached VO_2_ peak criteria [plateau in VO_2_, respiratory exchange ratio ≥ 1.1, HR approaching the age-predicted max, or volitional fatigue (high RPE)] or if there were clinical indications to terminate the test prior to reaching these criteria. VO_2_ peak was defined as the highest value in a 15 s epoch during the last stage of the test.

#### 2.4.3. Walking

- **10-meter walk test (10MWT).** Participants performed 2–3 trials of the 10MWT at comfortable and maximal speeds (van Bloemendaal et al., 2012). Time to complete the middle 6 m of the test was recorded with a stopwatch. Participants were permitted to use assistive devices if needed.
- **6-minute walk test (6mWT).** Participants walked as far as possible within 6 min, and the distance walked in meters was recorded as a measure of walking endurance (van Bloemendaal et al., 2012).

#### 2.4.4. Fatigue

- **Fatigue Severity Scale (FSS).** The FSS is a valid and recommended questionnaire that measures the severity of fatigue and effect on activities of daily living (Fisk et al., 1994; Krupp et al., 1989; Learmonth et al., 2013; Taskforce, 2011). Ratings across the 9 questions were averaged to get a final score.

#### 2.4.5. Cognitive function

- **Symbol Digit Modalities Test (SDMT):** The SDMT is a sensitive, valid, and reliable oral test that measures visual information processing speed (Benedict et al., 2017; Langdon et al., 2012). Participants performed as many symbol digit pairings as possible for 90 s. The total number of correct symbol digit pairings was recorded.
- **California Verbal Learning Test Second Edition (CVLT-II):** The CVLT-II is a valid and reliable measure of verbal learning and memory (Langdon et al., 2012; Stegen et al., 2010). Participants recalled as many words as possible from a list of 16 words. This was repeated 5 times, and the total recalled words across all trials was recorded.

### 2.5. Statistical analysis

Descriptive statistics included the mean (standard deviation) and range of scores, unless otherwise noted. No variables violated assumptions of normality or equality of variances between groups. Independent samples *t*-tests were used to compare groups for: 1) baseline characteristics; 2) session and training variables across all sessions (12 per participant); and 3) percent change in fitness, walking, fatigue, and cognitive outcomes. Cohen’s *d* was calculated to quantify effect sizes for changes per condition. Because the primary focus of this study was to determine the feasibility and safety of PVIT in this population, we elected to use parametric statistics despite the small sample size. However, for transparency, we also report the equivalent non-parametric statistics in the text (Mann Whitney U). Statistical analyses were performed in SPSS Statistics 25 (IBM, Armonk, NY, USA), with two-sided statistical testing with α = 0.05.

## 3. Results

PVIT and MICT groups did not differ in age, time since diagnosis, distributions of sex, race, ethnicity, more affected side, MMSE, or PDDS (Table 1, *p* ≥ 0.20). There were no adverse events.

### 3.1. Feasibility

#### Process feasibility:

**Fig. 2.**
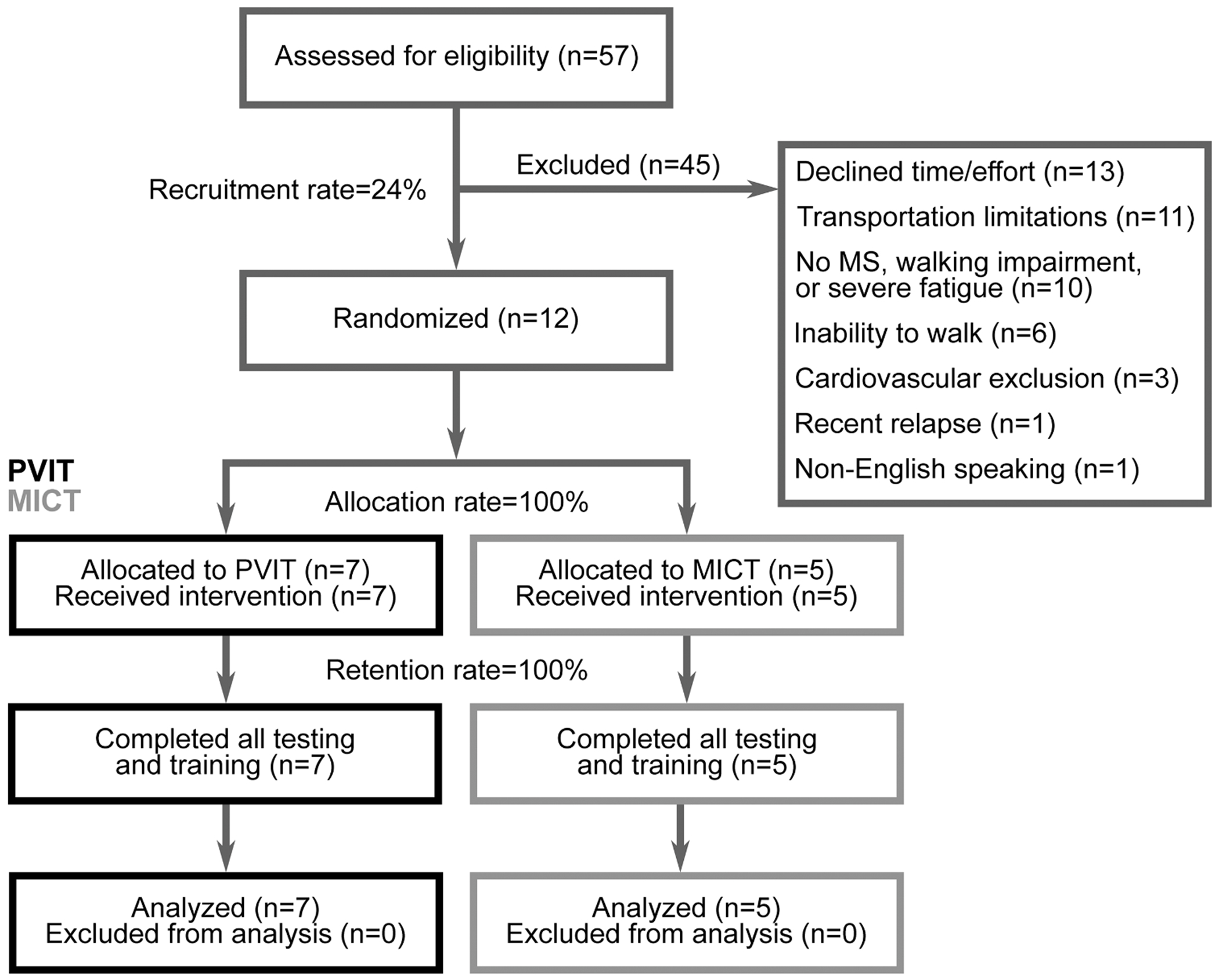
Flow diagram. Participant flow through the trial is shown, including recruitment rate, reasons for study exclusion, allocation rate, and retention rate. MICT: moderate-intensity continuous training. PVIT: peak velocity interval training.

A participant flow chart is shown in Fig. 2. 57 people were contacted for the study, and 12 were enrolled (recruitment rate = 24 %). All participants who enrolled were randomized successfully (randomization rate = 100 %).

#### Resource feasibility (Table 2):

All participants completed all testing and allocated training sessions (retention rate = 100 %). On average, it took participants ~38 days (~5.5 weeks range: 24–63 days) to complete the training with ~2 (range: 0–8) rescheduled sessions. Overall adherence was 86.1 (18.9) %, and did not differ between groups. All but one participant in the PVIT group had ≥75 % adherence. The overall duration compliance rate was 60 % and did not differ between groups. In each group, 3 participants had 100 % duration compliance. All participants had 100 % intensity compliance, regardless of meeting the duration prescription.

#### Intervention fidelity (Table 2):

**Fig. 3.**
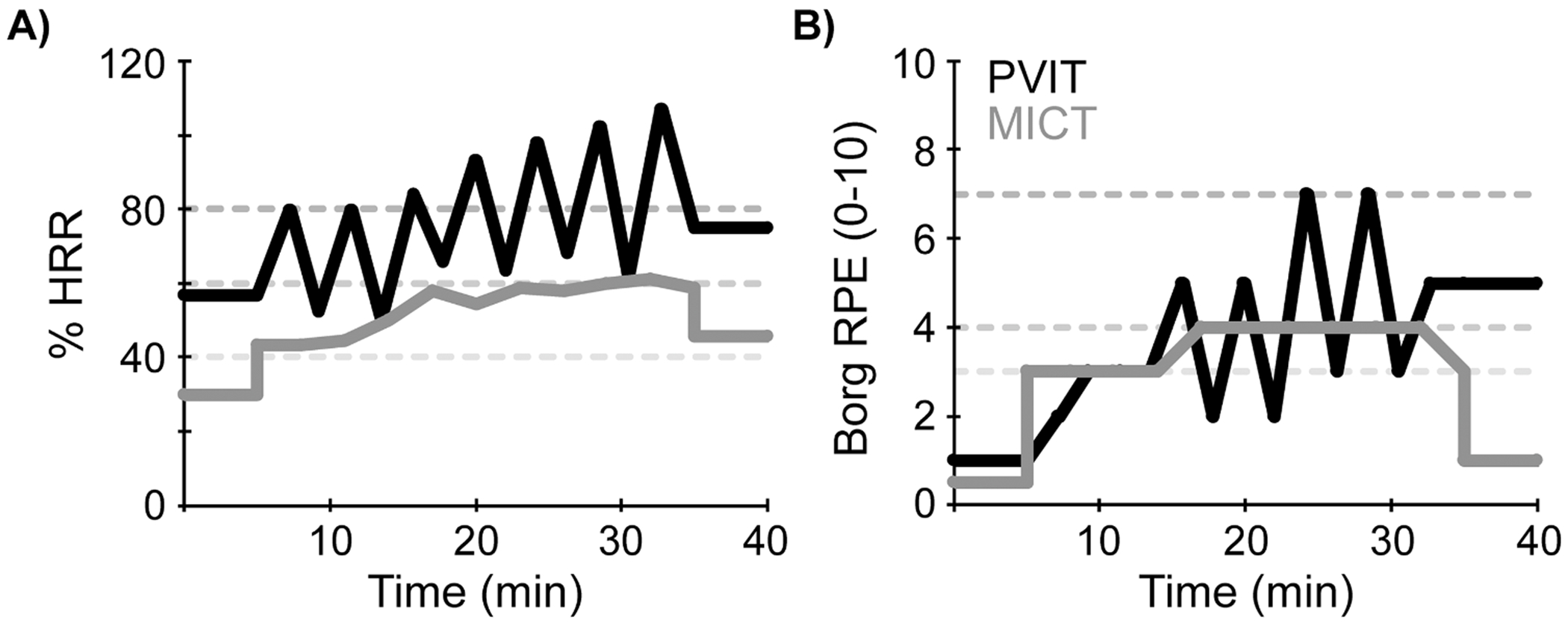
Example HR and RPE data. Data are shown from a single session in a single participant from the peak velocity interval training (PVIT) and moderate-intensity continuous training (MICT) groups. A) % Heart rate reserve (HRR) and B) Borg Rating of Perceived Exertion (RPE, 0–10 scale) are shown throughout the session for both groups. Dashed gray lines represent criteria for moderate intensity (40–60 % HRR and RPE of 3–4) and high intensity (>60 % HRR and RPE of 5+). Note that the individual in the PVIT group attained higher relative HR and RPE than the individual in the MICT group.

Although the high-intensity training was based on maximal walking speed and not HR, 92 % of intervals in the PVIT group still achieved HRs corresponding to vigorous physical activity (>60 % HRR). As expected, relative HR (%HRR) was significantly higher in the PVIT than the MICT group. Example HR and RPE data from one participant and session are shown in Fig. 3. On a whole session level, the PVIT group walked for less time per session than the MICT group (6.7 min less), because of a greater number of rests/pauses (2.5 min more) and early stoppage (see duration compliance above). The PVIT group walked for less distance than the MICT group (>800 m less). Peak velocity during intervals/segments did not differ between groups, but as expected, peak velocity relative to maximal overground velocity was greater in the PVIT than the MICT group, (95 % vs. 70 %).

### 3.2. Fitness, walking, fatigue, & cognitive function (Table 3)

**Fig. 4.**
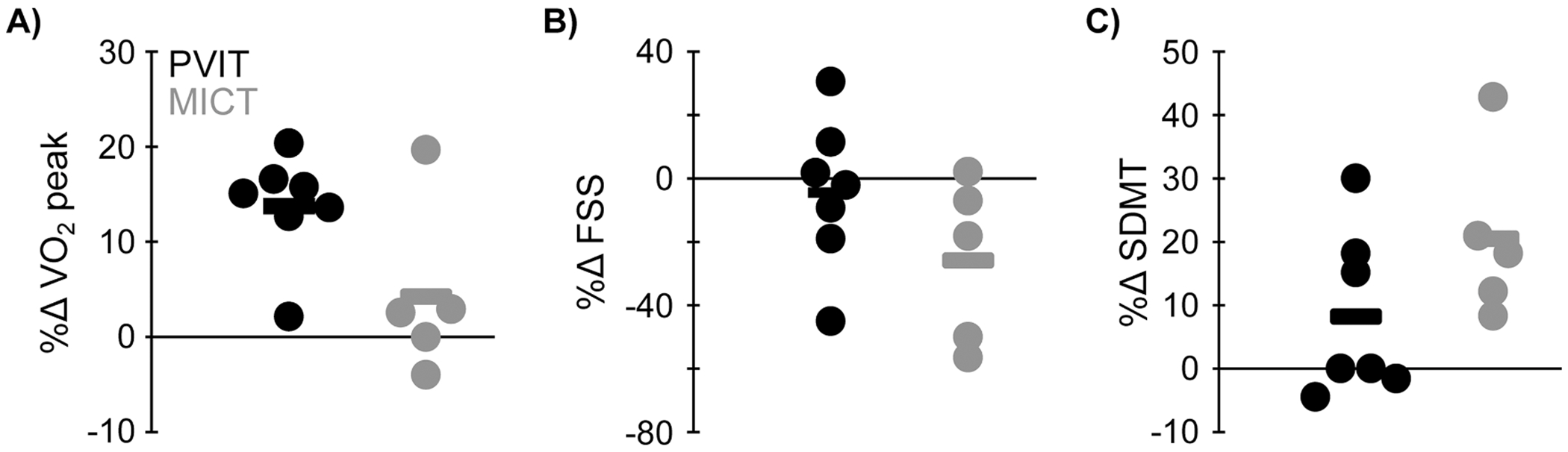
Group changes in primary outcome measures. Data show percent changes from pre to post in the primary outcome measures A) VO_2_ peak [peak oxygen consumption], B) FSS [Fatigue Severity Scale], and C) SDMT [Symbol Digit Modalities Test]. Each dot represents one participant in the peak velocity interval training (PVIT, black) and moderate-intensity continuous training (MICT, gray) groups. Horizontal bars represent group means. Data are jittered along the x-axis to avoid overlap.

VO_2_ peak improved by 2.3 mL/kg/min (14 % increase) in the PVIT group, compared with 0.4 mL/kg/min (1 % increase) in the MICT group [t(10) = 2.2, *p* = 0.05; *Z* = 1.5, *p* = 0.15]. There were no differences between groups in changes of any of the other outcomes (Fig. 4, t ≤ 1.9, *p* ≥ 0.09; *Z* ≤ 1.9, *p* ≥ 0.07).

## 4. Discussion

This pilot study examined the feasibility and preliminary efficacy of PVIT, a form of high-intensity walking training, in people with MS who had walking impairment and symptomatic fatigue. This approach is notable because few studies have investigated high-intensity *walking* in people with MS or included people with walking dysfunction and symptomatic fatigue. Results from this study suggest that PVIT is feasible in this subset of people with MS. This preliminary evidence further suggests that PVIT may yield greater effects on aerobic fitness than a traditional MICT approach. Such results may inform the use of high-intensity approaches in the MS population.

### 4.1. Feasibility

We report evidence that PVIT is feasible in people with MS who have walking dysfunction and symptomatic fatigue. All enrolled participants were successfully randomized, received the allocated intervention, and completed all aspects of the study (testing and training). Although we aimed to complete 12 training sessions in 4 weeks (3 sessions/week), most participants required more flexibility and completed the training in ~5 weeks. This longer duration was required because some participants were only scheduled for 2 sessions per week (not captured with the adherence measurement), and participants had on average 1–2 rescheduled sessions (86 % adherence). The choice was made to accommodate this variability in weekly exercise dose to enhance adherence (only scheduling 2 sessions per week) and accommodate fluctuating symptoms of MS or other real-life flexibility (allowing rescheduling). Differences in weekly exercise dose limit the methodological rigor of this study and may have affected the outcome measurements. At the same time, flexible scheduling likely enhances the generalizability and real-world applicability of the results.

Although target walking duration was 40 min, some participants could not attain this target, and the PVIT group walked for ~6.7 min less than the MICT group. A portion of this (2.5 min, 37 %) was related to the PVIT protocol, which requires pauses/rests if HR does not return near baseline during recovery segments. The remainder of this difference was likely driven by two participants in the PVIT group who averaged ~20 min or less of walking per session. Overall, duration compliance was 60 %. Although duration compliance was not statistically different between groups, a walking duration of 40 min may be less feasible for high compared to moderate intensity. However, despite this difference in training duration, the PVIT group still achieved greater improvements in aerobic power than the MICT group. Both groups had 100 % intensity compliance.

PVIT also had high intervention fidelity. The PVIT group achieved higher relative HR and walking velocities than the MICT group, and 92 % of HR values were within the target range. This is notable because the PVIT protocol focuses on achieving peak walking velocity and does not specifically target HR. It is also notable that participants were able to achieve higher HRs despite mobility impairments that may limit walking speeds.

These results align with other recent work supporting the safety and feasibility of high-intensity aerobic exercise interventions in people with MS, including balance training with high-intensity running/walking (Arntzen et al., 2023), cycle ergometry (Guillamó et al., 2018; Humphreys et al., 2022), recumbent stepping (Silveira et al., 2024), and arm ergometry (Skjerbæk et al., 2014). In the current study, results were within the range of values in these other feasibility studies: retention (current: 100 %, previous: 79–100 %), adherence (current: 86 %, previous: 30–97 %), and compliance (current: 100 %, previous: 96–100 %) The current work adds to the literature by establishing feasibility of high-intensity exercise training with a walking modality in people with MS with symptomatic fatigue and walking dysfunction. Prior work has not contained each of these components in one investigation.

### 4.2. Aerobic fitness, walking, fatigue, & cognitive function

Regarding the outcomes, we only report group differences for VO_2_ peak, which improved to a greater extent in the PVIT than the MICT group. This finding is consistent with a recent systematic review and meta-analysis wherein HIIT elicited greater improvements in aerobic capacity than MICT in people with MS (Youssef et al., 2024). This meta-analysis found a standardized mean difference across studies for VO_2_ peak of 0.45. In the current study, the between group effect size of change in VO_2_ peak exceeded this value (Cohen’s *d* = 0.86), indicating a greater effect than this prior work. Additionally, the change in VO_2_ peak for the PVIT group [2.3 (1.2) mL/kg/min; 13.8 (5.7)] was on the higher end of the range of effects found in previous studies involving high-intensity aerobic exercise training in people with MS (0–4.1 mL/kg/min; 0–18 %) (Feltham et al., 2013; Guillamó et al., 2018; Keytsman et al., 2021; Skjerbæk et al., 2014; Wens et al., 2015; Zimmer et al., 2018). The current results expand these findings to a walking modality and among individuals with walking impairment and symptomatic fatigue. One reason for the superiority of PVIT for improving VO_2_ peak may be that the repeated vascular stress and recovery periods of HIIT increases capacity for aerobic metabolism in type I fibers, capillarization, and oxidative enzyme activity more than MICT (Gibala et al., 2012; Laursen and Jenkins, 2002). There were some indications that walking speed may improve to a greater extent in the PVIT than the MICT group, although this did not reach statistical significance.

In contrast, fatigue and cognition changes trended toward greater improvements in the MICT group (again not statistically significant). The lack of statistically significant difference between PVIT and MICT is consistent with a recent meta-analysis (Youssef et al., 2024). However, because of the tendency for greater improvements in fatigue and cognition in the MICT group than the PVIT group, this is an important area of consideration for further research of HIIT interventions in people with MS. Perhaps among people with fatigue, high-intensity interventions may elicit greater acute exacerbations of symptomatic fatigue and impair cognition. Some studies have reported that greater symptomatic fatigue is associated with greater impairments in cognition (Eizaguirre et al., 2020).

### 4.3. Clinical implications

As demonstrated, PVIT or other HIIT approaches yield greater improvements in aerobic capacity than MICT, but there are some important considerations for clinical application. First, the PVIT group had greater improvements in aerobic capacity, despite the shorter session duration. Hence, these results suggest that PVIT or other HIIT approaches are more efficient approaches than MICT, supporting clinical utility. Moreover, although enjoyment was not assessed in this study, previous work has shown that HIIT is perceived as more enjoyable than MICT (Thum et al., 2017) .

Several future directions exist to expand this work (beyond a greater sample size). Future work should examine acute changes in fatigue following PVIT, as PVIT may yield greater increases in symptomatic fatigue than MICT. This could potentially lead to greater cognitive deficits, as discussed above. Additionally, greater symptomatic fatigue following PVIT could lead to decreases in daily physical activity during the training period. Future work also could use a mixed approach that combines aspects of moderate- and high-intensity exercise. This could consist of alternation of moderate- and high-intensity sessions or approaches like using moderate intensity to achieve a particular level of fitness before progressing to high-intensity exercise.

### 4.4. Study limitations

The primary weakness of this pilot study is the small sample size. The small sample size led to a reduced statistical power to detect smaller effects and also limits the generalizability of the findings to the broader population. Clearly, application to a greater number of participants is necessary to fully establish safety, feasibility, and efficacy. Although there were no baseline differences between groups, these were likely hard to detect because of the small sample size. Furthermore, there were trends that may have affected the results. Specifically, the PVIT group had a trend for a lower baseline VO_2_ peak (~4.4 mL/kg/min lower) and lower baseline walking speeds (~0.22 m/s and ~0.24 m/s slower for comfortable and maximal walking speeds). These baseline differences may have allowed for greater changes in some of the outcome variables in the PVIT group, although it is notable that baseline VO_2_ peak and walking speeds were not correlated with changes in these variables. A related issue is that we decided to use parametric statistics despite the small sample size. Non-parametric tests (presented above) were not significant for VO_2_ peak, indicating that these results should be interpreted with caution.

## 5. Conclusions

In people with MS who had walking dysfunction and symptomatic fatigue, this project: 1) supported the feasibility of PVIT by finding high rates of randomization, retention, adherence, and compliance, and 2) determined the preliminary efficacy of PVIT for improving aerobic fitness, walking, symptomatic fatigue, and cognition.

## Figures and Tables

**Table 1:** Demographics. Demographic and clinical characteristics of the peak velocity interval training (PVIT) and moderate-intensity continuous training (MICT) groups.

Total (*n* = 12)	PVIT (*n* = 7)	MICT (*n* = 5)
Age [years, mean (SD)]	48.6 (7.4)	50.4 (9.8)
Sex (male/female, counts)	2/5	1/4
Race (white/black/more than one)	2/4/1	4/1/0
Ethnicity (not Hispanic/Hispanic)	6/1	5/0
More affected limb (left/right, counts)	4/3	1/4
Years since diagnosis [mean (range)]	10.1 [1–24]	15.0 [5–34]
MMSE [0–30, mean (range)]	28.7 [26–30]	29.2 [29–30]
PDDS [0–8, mean (range)]	4.3 [3–7]	3.4 [3–4]

MMSE: Mini-Mental State Exam. PDDS: Patient-Determined Disease Steps. SD: standard deviation.

**Table 2:** Feasibility. Comparison of training for the peak velocity interval training (PVIT) and moderate-intensity continuous training (MICT) groups.

	PVIT	MICT	t	p
*Session overall*				
Retention (%)	100	100		
Time to complete training (days)	36.3 (13.8)	40.0 (8.8)	0.5	0.61
Rescheduled/cancelled sessions	1.9 [0–8]	1.4 [0–3]	0.3	0.75
Adherence (%)	84.5 (24.3)	88.3 (9.5)		
Session duration (min) [excludes rests or early stoppage]	30.4 (10.0)	37.1 (5.6)	4.7	**<0.001**
Duration compliance (%)	50 (47.9)	75 (37.3)	1.0	0.35
Intensity compliance (%)	100	100		
Pause/rest duration (min)	3.6 (3.7)	1.1 (1.8)	4.6	**<0.001**
Distance covered (m)	1285 (780)	2167 (1085)	6.1	**<0.001**
Peak interval/segment velocity (m/s)	1.04 (0.49)	1.06 (0.48)	0.2	0.83
Peak interval/segment velocity (% maximum overground velocity)	95 (41)	70 (12)	4.6	**<0.001**
*Across all intervals/segments*				
HR (bpm)	122.7 (13.1)	112.8 (8.1)	1.5	0.17
HR (%HRR)	82.6 (7.8)	55.5 (14.1)	2.2	**0.002**
HR (% in target range)	91.9 (9.7)	78.1 (15.7)	1.8	0.09
RPE (Borg 0–10)	4.4 (1.6)	3.9 (0.8)	0.7	0.48

HR: heart rate. HRR: heart rate reserve. RPE: rating of perceived exertion.

**Table 3:** Efficacy. Changes in outcomes from pre-to-post testing sessions in the peak velocity interval training (PVIT) and moderate-intensity continuous training (MICT) groups. Statistics are independent samples *t*-test comparing the % Δ in each variable. Bold p-values indicate statistical significance at *p* < 0.05.

		Pre	Post	Post-Pre (absolute Δ)	Post-Pre (% Δ)	Cohen’s d	Group	
							t	p
VO_2_ peak (mL/kg/min)	PVIT (*n* = 7)	16.6 (4.5)	18.9 (5.4)	2.3 (1.2)	13.8 (5.7)	0.47	2.3	**0.05**
	MICT (*n* = 5)	21.0 (3.1)	21.4 (5.5)	0.4 (2.9)	1.0 (12.9)	0.10		
c10MWT (m/s)	PVIT (*n* = 7)	0.89 (0.35)	1.01 (0.42)	0.11 (0.22)	12.5 (26.3)	0.30	1.0	0.34
	MICT (*n* = 5)	1.12 (0.31)	1.13 (0.40)	0.02 (0.10)	0.1 (8.8)	0.05		
m10MWT (m/s)	PVIT (*n* = 7)	1.25 (0.59)	1.33 (0.64)	0.08 (0.15)	5.7 (9.9)	0.13	0.9	0.38
	MICT (*n* = 5)	1.49 (0.44)	1.51 (0.56)	0.03 (0.16)	0.1 (11.1)	0.05		
6MWT (m)	PVIT (*n* = 7)	276 (148)	279 (143)	2.8 (29.7)	3.6 (13.3)	0.02	0.4	0.72
	MICT (*n* = 5)	365 (109)	397 (157)	31.8 (54.8)	6.5 (13.6)	0.24		
FSS [1–7]	PVIT (*n* = 7)	5.5 (1.0)	5.1 (1.2)	−0.4 (1.4)	−4.5 (23.8)	0.34	1.5	0.17
	MICT (*n* = 5)	5.5 (0.6)	4.1 (1.6)	−1.4 (1.4)	−25.9 (26.1)	1.30		
SDMT [0–110]	PVIT (*n* = 7)	40.3 (18.8)	41.9 (15.9)	1.6 (3.4)	8.2 (13.0)	0.09	1.6	0.14
	MICT (*n* = 5)	44.0 (7.4)	53.8 (15.6)	9.8 (8.3)	20.5 (13.4)	0.85		
CVLT-II [0–80]	PVIT (*n* = 7)	37.9 (14.6)	37.0 (13.7)	−0.9 (6.4)	−1.5 (16.9)	0.06	1.9	0.09
	MICT (*n* = 5)	40.8 (6.4)	47.8 (8.1)	7.0 (8.9)	18.7 (20.8)	0.97		

6MWT: 6-minute walk test. c10MWT: comfortable 10-meter walk test. CVLT-II: California Verbal Learning Test Second Edition. FSS: Fatigue Severity Scale. m10MWT: maximal 10-meter walk test. SDMT: Symbol Digit Modalities Test. VO_2_ peak: peak oxygen consumption.

## Data Availability

Upon publication, data for this study will be openly available at: doi.org/10.25417/uic.28661129. https://figshare.com/s/246ecd09767221326d65

## Glossary

10MWT: 10-meter walk test
6mWT: 6-minute walk test
BP: blood pressure
CVLT-II: California Verbal Learning Test Second Edition
FSS: Fatigue Severity Scale
HIIT: high-intensity interval training
HR: heart rate
HRR: heart rate reserve
MICT: moderate-intensity continuous training
MMSE: Mini Mental State Examination
MS: multiple sclerosis
PDDS: Patient-Determined Disease Steps
PVIT: peak velocity interval training
RPE: Rating of Perceived Exertion
SDMT: Symbol Digit Modalities Test
UIC: University of Illinois Chicago
VO_2_ peak: Peak rate of oxygen consumption
